# Few-qubit quantum refrigerator for cooling a multi-qubit system

**DOI:** 10.1038/s41598-021-92258-0

**Published:** 2021-06-21

**Authors:** Onat Arısoy, Özgür E. Müstecaplıoğlu

**Affiliations:** 1 Institute for Physical Science and Technology, University of Maryland, College Park, MD 20742 USA; 2 Department of Physics, Koç University, 34450 Sarıyer, Istanbul Turkey

**Keywords:** Statistical physics, Qubits, Quantum simulation

## Abstract

We propose to use a few-qubit system as a compact quantum refrigerator for cooling an interacting multi-qubit system. We specifically consider a central qubit coupled to *N* ancilla qubits in a so-called spin-star model to be used as refrigerant by means of short interactions with a many-qubit system to be cooled. We first show that if the interaction between the qubits is of the longitudinal and ferromagnetic Ising model form, the central qubit is colder than the environment. We summarize how preparing the refrigerant qubits using the spin-star model paves the way for the cooling of a many-qubit system by means of a collisional route to thermalization. We discuss a simple refrigeration cycle, considering the operation cost and cooling efficiency, which can be controlled by *N* and the qubit–qubit interaction strength. Besides, bounds on the achievable temperature are established. Such few-qubit compact quantum refrigerators can be significant to reduce dimensions of quantum technology applications, can be easy to integrate into all-qubit systems, and can increase the speed and power of quantum computing and thermal devices.

## Introduction

The widespread use of quantum technologies is limited by the large and expensive cooling systems required for their implementations. The rapidly emerging field of quantum thermodynamics^1–5^ paves the way for compact, fast, and efficient quantum refrigeration schemes for quantum devices^6,7^. Pioneering studies are limited to cooling a single two-level system (qubit or spin-1/2 particle)^8^. A critical question for practical quantum machines is if and how such quantum refrigerators can cool down interacting multiple qubit systems. We propose a few-qubit quantum refrigerator with scalable advantages in its cooling efficiency and achievable minimum temperatures as a possible positive answer to this question.

Early quantum refrigerator studies consider utilization of quantum coherence injected by external drives^8^, spectral bath filtering and periodically modulated interactions^9^, and frequent measurement schemes^10^. Intriguing proposals based on quantum coherence and entanglement to cool quantum systems^11,12^ are proposed. Another recent work^13^ discusses the possibility of exploiting many-body localized (MBL) states of large quantum systems for scalable engines and refrigerators in the quantum regime. A more conventional cooling method for spin systems is known as algorithmic cooling^14–16^. How it can be part of a quantum algorithmic heat engine has been recently presented^17^. Continuous variants of algorithmic cooling a single qubit or an oscillator using three-body interactions are explored^18–22^ and experimentally demonstrated in trapped ion systems^23^. A three-qubit system with a tunable engine or refrigerator operation using only two-qubit interactions is recently investigated^24^. However, more progress in this field is still well desirable to generalize these refrigerators from single qubits or harmonic oscillators to quantum many-body systems. Doing this properly requires some caution as all of the aforementioned refrigerator ideas rely on different elements of non-equilibrium dynamics. Therefore, their generalization to the many-body thermalization needs to be scrutinized about the existence and uniqueness of an equilibrium state and whether it is a thermal state for an effective temperature instead of a NESS.

**Figure 1:**
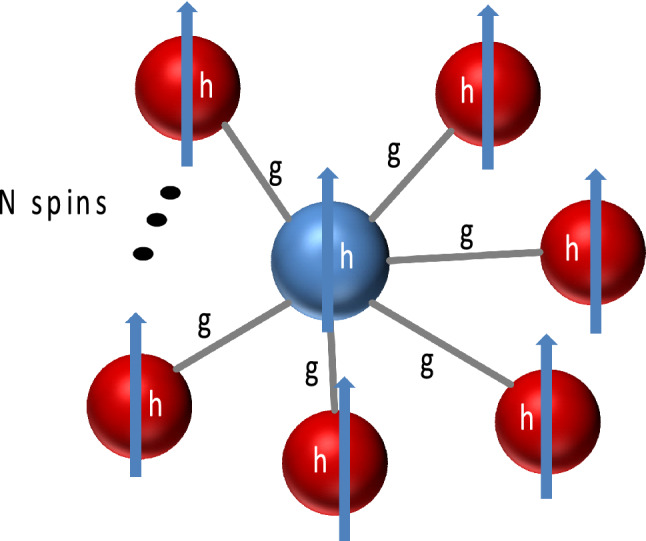
Sketch of spin-star model (system A) consisting of a central spin-1/2 particle (blue sphere) surrounded by *N* spin-1/2 particles (red spheres). Central spin (system B) is coupled to the surrounding spins with the same interaction coefficient *g*. The whole system is in a homogeneous magnetic field *h*. We assume the interactions only contains longitudinal spin components, in the same direction with the magnetic field. The spin-star model is used to describe a $(N+1)$-qubit quantum refrigerator, where each spin effectively represents a qubit with an energy gap *h*. When the system is in thermal equilibrium with an environment at temperature *T*, the central qubit is in a Gibbsian state with an effective a temperature smaller than *T*. The central qubit can be used as the refrigerant to cool other quantum many-body systems (cf. Fig. 4).

Recently, a scheme, closely related to the algorithmic cooling idea of entropy transfer among qubits, to thermalize a many-body system by repeated collisions has been proposed^25^ as a generalization of earlier works on thermalization of single body systems, typically a quantum harmonic oscillator or a single qubit, with collisions^26,27^. Collision models allow for microscopic surgery of a wide range of environment models, including the non-Markovian ones^28^, but we limit ourselves here to the case of Markovian dynamics^25,29^. The random collisions are one of the oldest routes considered for describing thermalization, introduced by Lord Rayleigh^30^. A massive particle thermalizes after many random collisions by small projectiles in thermal states. This mechanism explains the micromaser in the blackbody radiator regime, where the optical cavity is heated by thermal pump atoms^31^. More recent studies showed that pump atoms in quantum coherent states could also be used to heat the micromaser^32–34^. A particularly intriguing scenario is the scalable heating of the micromaser with the number of pump atoms, using a so-called spin-star system^34^. Spin-star configuration consists of a central qubit surrounded by *N* ancilla qubits (cf. Fig. 1). The critical point is that the central qubit can be at a higher local temperature than the environment.

This paper shows that when the central spin model contains only the longitudinal spin components and ferromagnetic interactions, the central spin becomes locally colder than the environment. The longitudinal spin-star model defined in the next section can be treated with the usual methods of classical statistical mechanics, and hence possible quantum advantages in engines and refrigerators^11,12,18–23,35,36^ will be out of the scope of present work, except a brief discussion of the Heisenberg spin-star model in the SI appendix. The $(N+1)$-qubit system can be envisioned as a quantum refrigerator, where the central qubit can be used as a quantum refrigerant to cool other systems, specifically, an interacting multi-qubit system. For that aim, it is necessary to contact the quantum refrigerant with the many-body system. The required refrigerant-system coupling can be performed within the collisional route to many-body thermalization^25^. Successful coupling needs matching refrigerant frequencies to transition frequencies of the many-body system. Therefore, our proposal can be envisioned as an all-qubit network with integrated quantum refrigerators (cf. Fig. 4). We offer a three-step study of this refrigeration setup: first, we study the energy cost and efficiency of the preparation of the refrigerant qubits; then we discuss the repeated interactions between the refrigerant qubits and the target many-body system and we conclude our study by proposing two different ways to improve the efficiency of the refrigeration cycle.

Finally, we should clarify the similarities with the algorithmic cooling. There is only a single bath (environment) where the spin-star qubit structure is held. Such quantum “molecule” has a central qubit at a local thermal equilibrium colder than the environment due to longitudinal ferromagnetic qubit-qubit “bonding”. While the initial thermal states’ preparation is relatively easy in our scheme, we still need resetting and timing control in the quantum cooling network. Similar to algorithmic cooling, timing and control can be achieved by using qubits at different thermalization rates. Another significant advantage here is to have a readily integrable few-qubit refrigerator with a single qubit refrigerant for compact, fast, and efficient cooling of a many-qubit system.

## Model system for the preparation of the refrigerant qubits

We consider a so-called “spin-star” system consisting of a single qubit surrounded by *N* ancilla qubits, as illustrated in Fig. 1. For the sake of clarity in what the word “system” refers to in the rest of the text, we will denote the whole spin-star system as system A, its central spin as system B and the many-body system to be cooled as system C. Interactions between the central qubit and the surrounding qubits are assumed to be the same, characterized by the coupling coefficient *g*. The energy gap of the qubit is denoted by *h*. We represent each qubit as an effective spin-1/2 particle and further assume that the qubit-qubit interactions are only between the *z*-components of the effective spins. The specification of interaction direction is neither for simplicity nor arbitrary. Transverse components cause correlations and entanglement in the eigenstates of the Hamiltonian, which is not desirable for our purpose of cooling the system. Further explanation of the harmful influence of transverse interactions on cooling is given in the SI appendix. The total Hamiltonian of system A can be written as

1 $$\begin{aligned} {\hat{H}} = h\sum _{n=0}^{N}{\hat{\sigma }}_{z,n} + g~{\hat{\sigma }}_{z,0}\sum _{n=1}^{N}{\hat{\sigma }}_{z,n}, \end{aligned}$$

where the indices $n=0$ and $n=1,2\dots N$ indicate the central qubit and surrounding qubits, respectively. ${{\hat{\sigma }}_{z,0}},{{\hat{\sigma }}_{z,n}}$ are the *z*-component Pauli spin operators.

As the Pauli spin operators are only for the *z*-components, the model can be considered a longitudinal Ising model^37^, but with a spin-star configuration instead of a spin chain. Spin-star models are special cases of Richardson–Gaudin models, which are usually studied in the context of hyperfine interactions in semiconductor quantum dots^38,39^ and as a toy model of non-Markovianity^40–42^. However, the semiconductor quantum dot implementation of spin-star models will not be relevant for our purposes. It is based on Heisenberg interactions, which we discuss and rule out for our purposes in the SI appendix. For a superconducting qubit implementation of our proposal, a generalization and re-configuration of the Chimera unit cell architecture used in D-Wave quantum annealers seems possible. This architecture makes use of orthogonally placed qubits overlapping each other and allowing to set couplers between horizontal and vertical qubits, which generate a longitudinal Ising interaction^43^ while keeping undesired interactions at a level lower by 3 orders of magnitude than the controlled interactions with a careful engineering of mutual inductances between qubits and their components^44^.

## Results

In our numerical simulations, we will consider an artificial spin system, specifically a system of superconducting two-level systems (qubits). Efficient, compact, and fast cooling of such superconducting interacting qubits is a critical problem for practical quantum computations. Hence, we focus our range of parameters on this particular case, though our generic models, exact analytical results, and general conclusions apply a broader class of physical systems. We will call “effective spin,” representing a qubit as “spin” in the following discussions for brevity. We take $\hbar =1$ and set $h=1\text { GHz}$ for all of our calculations as it is a typical order of magnitude for superconducting qubits^45^ and we will assume that *g* can be in the order of *h*^46^.

### Thermal state for the spin-star model and effective temperature of the refrigerant qubit

The eigenstates of the Hamiltonian in Eq. (1) are not entangled. Off-diagonal elements of the total density matrix vanish in the tensor product of the z-basis of each effective spin. Accordingly, we can treat the Ising spin-star model as a classical discrete system (with up and down spin states labeled by $z=+1$ and $z=-1$, respectively) and study the state probability distribution described by the diagonal elements of the total density matrix.

We consider the spin-star system immersed in a thermal environment at temperature *T*. The thermal environment is usually the natural environment common to all spin-star system qubits and it is reasonable to assume that the interaction of the spin-star system with this environment cannot be tuned or interrupted at will. We can define the partition function of the whole system by treating up and down states of the central spin separately. Assuming the central spin is in the $z_0=\pm 1$ state, the partition function of a single ancilla spin equals to that of a non-interacting spin with Hamiltonian eigenvalue $h\pm g$. The partition function of all ancilla spins is obtained simply by taking $N{\text {th}}$ power of the partition function of a single ancilla. Summing the partition functions of ancilla spins for up (down) states of the central spin with factors $\exp {(-(+)\beta h)}$, we find the partition function of the system A to be

2 $$\begin{aligned} Z_{\text {tot}} = 2^{N}(e^{-\beta h}\cosh ^N(\beta (g+h))+e^{\beta h}\cosh ^N(\beta (h-g))){,} \end{aligned}$$

where $\beta = 1/k_B T$ with $k_B$ being the Boltzmann constant. We can find the reduced state of a subsystem by taking a partial trace over other subsystems. If this reduced state can be written in a canonical Gibbsian form, an effective local temperature can be assigned to the subsystem. For our spin-star system, the central spin subsystem is a qubit for which we can always find a Gibbsian state and a local temperature.

The first term of Eq. (2) corresponds to the up state of the central spin while the second corresponds to its down state. That remark allows us to give explicit expressions for the probabilities of the states of the central spin

3 $$\begin{aligned} P(z_0 = \pm 1) = \frac{2^N e^{\mp \beta h} \cosh ^N(\beta (h\pm g))}{Z_{\text {tot}}}. \end{aligned}$$

Knowing that the coherence terms of the density matrix of the central spin vanish after partial tracing of the total density matrix over ancilla spins, the effective (local) inverse temperature of the central qubit (system B) $\beta _\text {eff}$ as a function of its state populations is defined by

4 $$\begin{aligned} \beta _{\text {eff}} = \frac{1}{2h}\ln \left( \frac{P(z_0 =-1)}{P(z_0 =1)}\right) =\frac{1}{2h}\left( 2\beta h + N\ln \left( \frac{\cosh (\beta (h-g))}{\cosh (\beta (h+g))}\right) \right) = \beta + \frac{N}{2h}(\ln (\cosh (\beta (h-g)))-\ln (\cosh (\beta (h+g)))). \end{aligned}$$

**Figure 2:**
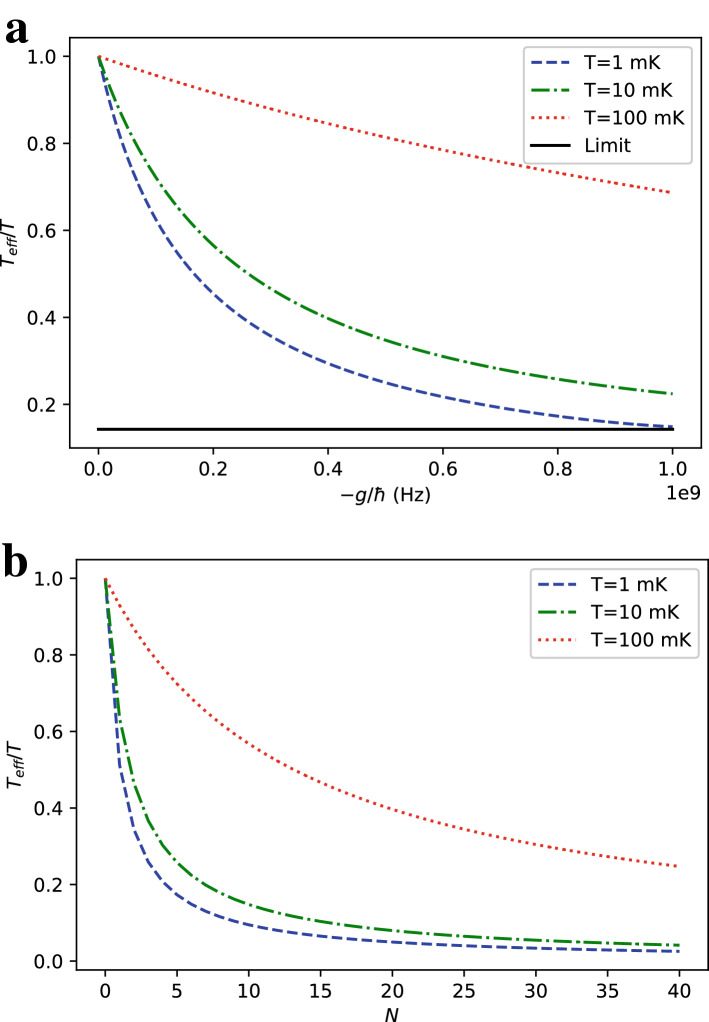
Ratio of the effective $T_\text {eff}$ temperature of the central qubit to the environment temperature *T* in a longitudinal ferromagnetic Ising spin-star model with $h=1$ GHz for (**a**) $N=6$ ancilla qubits at different interaction strengths *g*, (**b**) $g=-h$ at different number of ancilla qubits *N*.

Taking the derivative of $\beta _{\text {eff}}$ with respect to the interaction strength *g* here turns out to be insightful.

5 $$\begin{aligned} \frac{\partial \beta _{\text {eff}}}{\partial g} = \frac{-N\beta }{2h}(\tanh (\beta (h-g))+\tanh (\beta (h+g))) \end{aligned}$$

As $\tanh$ is a one-to-one odd function, setting the derivative to zero requires $h-g = -(h+g) = -h-g$, which is not satisfied for any value of *g*. Thus, $\beta _{\text {eff}}$ is a monotonic function of *g* and evaluating the derivative for $g=0$ further shows that $\beta _{\text {eff}}$ is a monotonically decreasing function of *g*. For our purposes, this guarantees $\beta _{\text {eff}} > \beta$ when $g<0$, proving that our proposed setup manages to cool down the central qubit for ferromagnetic type interactions. Also, by monotonicity of $\beta _{\text {eff}}$ as a function of *g*, it keeps increasing while *g* diverges towards $-\infty$, meaning that its limit at $-\infty$ is also its upper bound.

6 $$\begin{aligned} \beta _{\text {max}}= & {} \lim _{g \rightarrow -\infty } \beta _{\text {eff}} = \beta + \frac{N}{2h} \ln \left( \lim _{g \rightarrow -\infty } \frac{\cosh (\beta (h-g))}{\cosh (\beta (h+g))} \right) = \beta + \frac{N}{2h} \ln \left( e^{2\beta h} \right) = (N+1)\beta \end{aligned}$$

Figure 2 shows the ratio of the effective temperature $T_\text {eff}$ to the environment temperature *T* for different interaction strengths *g* and number of ancilla qubits *N*. The asymptotic theoretical limit of the $T_\text {eff}$ in Eq. (6) is approached faster with increasing *g* in the low *T* regime as shown in Fig. 2a. Figure 2b suggest that, towards $g\sim -h$, reasonably large values of *N* can achieve an order of magnitude cooling of the central qubit relative to typical environment temperatures in superconducting circuits ($<20$ mK).

As a side note, we generalize the analytical results of this section to a spin-star system with different interaction strengths while keeping the homogeneous longitudinal magnetic field strength on the qubits. Using the arguments we used to derive Eq. (2) for each environment qubit with a given interaction strength $g_i$, we obtain a more general expression which covers Eq. (2) when $g_i = g$.

7 $$\begin{aligned} Z_{\text {tot}} = 2^N (e^{-\beta h} \prod _{i=1}^N \cosh (\beta (g_i + h)) + e^{\beta h} \prod _{i=1}^N \cosh (\beta (h - g_i))) \end{aligned}$$

Thus, for non-uniform interaction strengths, the effective temperature calculated in Eq. (4) becomes

8 $$\begin{aligned} \beta _{\text {eff}} = \frac{1}{2h}\left( 2\beta h + \sum _{i=1}^N\ln \left( \frac{\cosh (\beta (h-g_i))}{\cosh (\beta (h+g_i))}\right) \right) = \beta + \frac{1}{2h} \sum _{i=1}^N\ln \left( \frac{\cosh (\beta (h-g_i))}{\cosh (\beta (h+g_i))}\right) . \end{aligned}$$

From this expression, it is straightforward to prove that cooling is achieved if all $g_i$ are negative and $\beta _{\text {eff}}$ is a monotonically decreasing function of all $g_i$. In other words, the equilibrium state of the spin-star system with different interaction strengths shows similar behavior with the one with identical interactions. However, unless some specific implementation of the model does not allow to engineer identical interactions, there is no particular benefit in considering different interaction strengths in our proposal and we will stick to identical interactions for the rest of the paper.

In what follows, we are going to discuss the energy cost and efficiency of the preparation of the central (refrigerant) qubits. Then, we are going to elaborate on the relevance of preparing the central qubits (system B) at a lower effective temperature in the context of a refrigerator for an interacting many-qubit system (system C) by means of a collisional route to thermalization^25^. We will describe a refrigeration cycle for the target many-qubit system by succesively turning on and off the coupling between refrigerant and ancilla qubits of the spin-star systems so that repeated interactions of the refrigerant qubit replicas with the many-qubit system describes irreversible dynamics for the target system in an effective cold environment according to collision model.

### A simple refrigeration cycle and its efficiency

In this section, we study the energy cost and efficiency of preparing the refrigerant qubits (system B) by considering a cyclic transformation of the whole spin-star system (system A) in a single thermal environment whose interactions with system A are not controllable, in other words, these interactions cannot be switched off or have time dependence. The cycle begins with uncoupled qubits ($g=0$) in thermal equilibrium at the environment temperature *T*.

In the first step, the longitudinal Ising interactions of system A is suddenly switched on so that there is no entropy change. At this stage, work is taken from the system, and there is no heat exchange with the environment as the duration of this stage is too short for the thermal environment, which is implicitly assumed to be weakly coupled to the system A, to have an effect on the system.

The interacting qubits (system A) are left to thermalize to *T* in the second step by means of its interactions with the environment at temperature *T*. While the system A is in thermal equilibrium with the environment at *T*, the system B is not. Its effective temperature is given by Eq. (4).

The third step consists of suddenly turning off the interactions within system A ($g\rightarrow 0$) such that the state of the central (refrigerant) qubit does not change, which is somehow similar to the idea of setting the expectation of the interaction Hamiltonian to zero by means of frequent measurements studied in a previous work^10^. Under this assumption the transitions and the associated changes in $T_\text {eff}$ are negligible. In general, preservation of the initial state under a sudden perturbation requires that the switching on or off the interaction must be much faster than any characteristic time scale of the system, which is 1/2*h* for the central qubit. This condition is relaxed in our case, as the longitudinal Ising interactions [cf. Eq. (1)] cannot cause any excitations in the initial thermal state before the quench. We can still introduce a bound on the perturbation time $\tau$. In practice, the qubits may not be uncoupled from the environment during the switching and hence we require $\tau \ll \tau _{\text {rel}}$ where $\tau _{\text {rel}}$ is the relaxation (thermalization) time of the central qubit. Hence the central qubit remains cold at $T_\text {eff}$ for a duration of $\tau$. This gives us a “cooling window” in which the central qubit (system B) can be used as a refrigerant to cool a many-qubit system (system C) by the collisional route to thermalization^25^, as described in the next section.

The fourth step makes use of the cooling window mentioned above for the interactions between the system C and the system B at its effective temperature $T_{\text {eff}}$ to make this setup a refrigerator for system C. However, this interaction will not change our following results for the efficiency of the cycle. After the interactions between systems B and C, this step ends with the thermalization of non-interacting central and ancilla qubits by the environment, bringing the whole system back to the beginning of the refrigeration cycle.

The cooling of the central qubit is performed with an efficiency which is conceptually defined as the ratio of the energy extracted from the central qubit to the work cost of the cycle. Here, we make an application oriented reasoning to come up with this efficiency definition and we need to emphasize that this definition is not unique. First, it is easy to notice that the energy extracted from system B in this cycle increases with decreasing effective temperature, so our efficiency definition reflects the trade-off between cooling system B to very low temperatures and its increasing energy cost. An efficiency definition based on the energy extracted from the system C by means of collisions is also questionable as it would also depend on the parameters of the collision model and the nature of the system C. Another possible choice of efficiency could use the energy extracted from the whole spin-star system (system A) rather than only the central spin (system B). However, it would not make sense to include the energy extracted from the ancilla qubits of system A as they regain that energy at the end of the refrigeration cycle and their cooling is not useful in the case of cooling of system C with only central spins, which is studied in this section. The use of ancilla spins for cooling of the system C will come up in the rest of the paper and the efficiency definition will be accordingly modified for that case.

We express the efficiency we define as

9 $$\begin{aligned} \varepsilon = \frac{E_s(\beta )-E_s(\beta _{\text {eff}})}{W_{\text {cycle}}} = \frac{h(\tanh (\beta _{\text {eff}} h) -\tanh (\beta h))}{W_{\text {cycle}}} \end{aligned}$$

where $E_s(\beta )$ is the expectation of the Hamiltonian of the system B at inverse temperature $\beta$ and $W_{\text {cycle}}$ is the net work cost of turning on and off the Ising interactions. The interaction of the system B with the system C at the end of the third step of the cycle does not affect the central qubit’s cooling efficiency.

To define the efficiency of the cycle, we need to calculate $W_{\text {cycle}}$ from the internal energy of the system A at the end of each step of the cycle. The total energy is given by

10 $$\begin{aligned} E_{0}=-(N+1)h\tanh (\beta h) \end{aligned}$$

at the beginning of the cycle. After sudden quench by turning on the interaction, the state of the central qubit is preserved while the energy change is equal to the expectation of the interaction Hamiltonian at the initial state. As the state probability distribution of each qubit is independent, the total energy at the end of the first step $E_{1}$ can be calculated as

11 $$\begin{aligned} E_{1}=-(N+1)h\tanh (\beta h) +\frac{gN(\cosh (2\beta h)-1)}{2\cosh ^2(\beta h)}. \end{aligned}$$

We can calculate the energy of the interacting system in thermal equilibrium at the end of the second step by using the partition function in Eq. (2).

12 $$\begin{aligned} E_{2}= & {} -\frac{\partial \ln Z_{\text {tot}}}{\partial \beta } \nonumber \\= & {} -\frac{e^{-\beta h}\cosh ^{N-1}(\beta (g+h))(N(g+h)\sinh (\beta (g+h))-h\cosh (\beta (g+h)))}{e^{-\beta h}\cosh ^N(\beta (g+h)) + e^{\beta h}\cosh ^N(\beta (h-g))} \nonumber \\&-\frac{e^{\beta h}\cosh ^{N-1}(\beta (h-g))(N(h-g)\sinh (\beta (h-g))+h\cosh (\beta (h-g)))}{e^{-\beta h}\cosh ^N(\beta (g+h)) + e^{\beta h}\cosh ^N(\beta (h-g))} \end{aligned}$$

Finally, we can calculate the total energy of the system, $E_{3}$ after turning off the interaction at the end of the third step by calculating the expectation of the interaction Hamiltonian and subtracting it from $E_{2}$.

13 $$\begin{aligned}&<{\hat{H}}_{\text {int}}> = \frac{-g}{\beta }\frac{\partial \ln Z_{\text {tot}}}{\partial g}=\frac{-gN(e^{-\beta h}\cosh ^{N-1}(\beta (g+h))\sinh (\beta (g+h))-e^{\beta h}\cosh ^{N-1}(\beta (h-g))\sinh (\beta (h-g)))}{e^{-\beta h}\cosh ^N(\beta (g+h)) + e^{\beta h}\cosh ^N(\beta (h-g))}\end{aligned}$$

14 $$E_{3} = E_{2} - <{\hat{H}}_{\text {int}}>$$

As Eqs. (12) and (14) are fairly long, we are not going to write down the explicit expression for the total work in a cycle and restrict ourselves to express it in terms of the energies at different stages of the cycle.

15 $$\begin{aligned} W_{\text {cycle}}= & {} W_1 + W_2 = (E_{1}-E_{0})+(E_{3}-E_{2}) = \frac{gN(\cosh (2\beta h)-1)}{2\cosh ^2(\beta h)} - <{\hat{H}}_{\text {int}}> \end{aligned}$$

**Figure 3:**
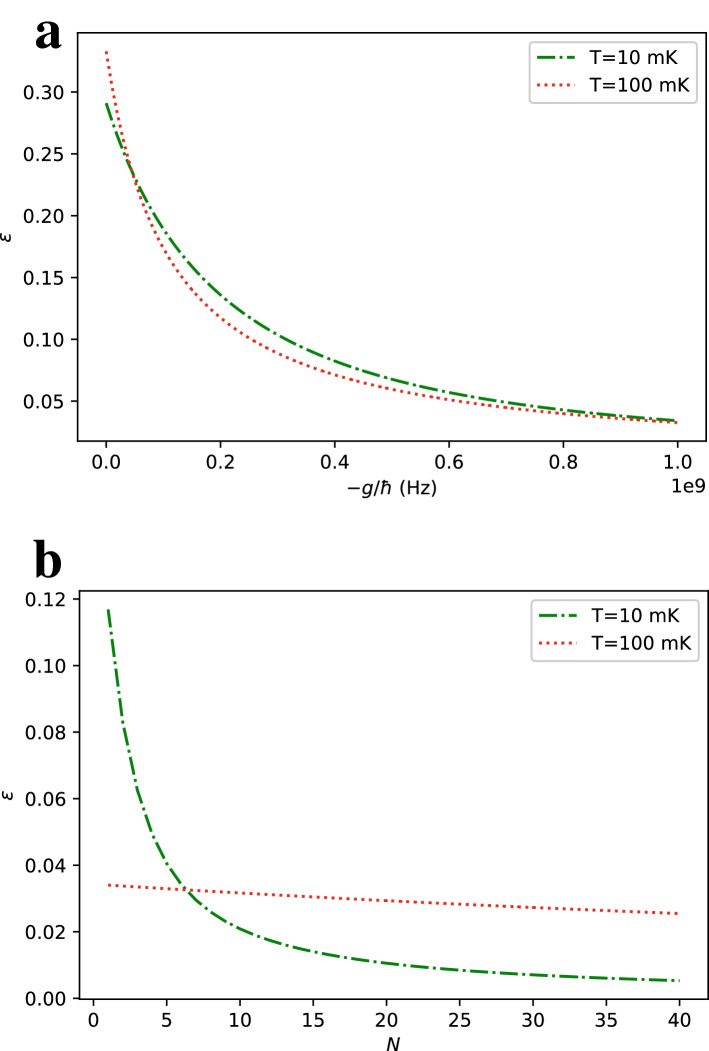
Efficiency $\varepsilon$ of the refrigeration cycle defined in Eq. (9) as a function of (**a**) interaction strength *g* with $N=6$ and (**b**) number of ancilla qubits *N* with $g=-h$ at different environment temperatures *T* and for $h=1$ GHz.

The resulting efficiency with different number of ancilla qubits *N* and different interaction strengths *g* are plotted in Fig. 3. Figure 3a indicates that efficiency decreases with *g*. Comparing with Fig. 2a, we deduce that cooling to lower temperatures with increasing *g* is not efficient. Similar conclusion can be made for cooling by increasing *N* after comparing Figs. 2b and 3b. An optimum strategy would be to use lower *g* and *N* values, relative to highest available ones, to cool to the target temperatures within acceptable efficiencies. For example, about an order of magnitude cooling can be achieved in typical superconducting qubit environment temperatures with $\sim 10\%$ efficiency for $g\sim -h/2$ and $N = 6$. In the last section before the discussion, we will discuss exploiting the ancilla qubits to further increase the efficiency of the cooling cycle. It is also worth mentioning that there is some room to optimize our cycle even without using the ancilla qubits by merging the last and first steps of the cycle with a time-dependent protocol for turning on the Ising interaction under the influence of the environment instead of separate thermalization and quenching steps. The effects of fast driving under non-Markovian open system dynamics has recently been studied in a quantum Stirling heat engine and an increase of efficiency is numerically demonstrated^47^. We could expect a similar efficiency improvement in our setup by extracting more work while turning on the interaction, however, we do not explore this path further in this paper as it requires knowledge about the nature of the interactions of the spin-star system with its thermal environment and the fine tuning of this protocol would intensively rely on numerical optimization based on the environment parameters.

### Cooling of a many-body system with spin-star quantum refrigerators

We start the discussion of quantum many-body system cooling by summarizing how a previous work on quantum many-body thermalization by a collision model^25^ allows the use of central qubits as refrigerants for a many-body system and we outline its findings in the SI appendix. The system qubits make repeated collisions with a set of “bath” qubits. The number of bath qubits depends on the number of transition frequencies of the many-body system. The scheme is suitable for cooling a small many-body system with a finite set of discrete eigenfrequencies in practice while being flexible for the cooling arbitrary sized qubit systems as the number of available qubits in a quantum system and the maximum possible number of simultaneous couplings to a single qubit increase with future technological improvements. As a reliable benchmark for the current limitations on simultaneous qubit couplings, we can take the Chimera unit cell architecture of D-Wave annealers where a qubit can simultaneously be coupled to four different qubits^43^.

**Figure 4:**
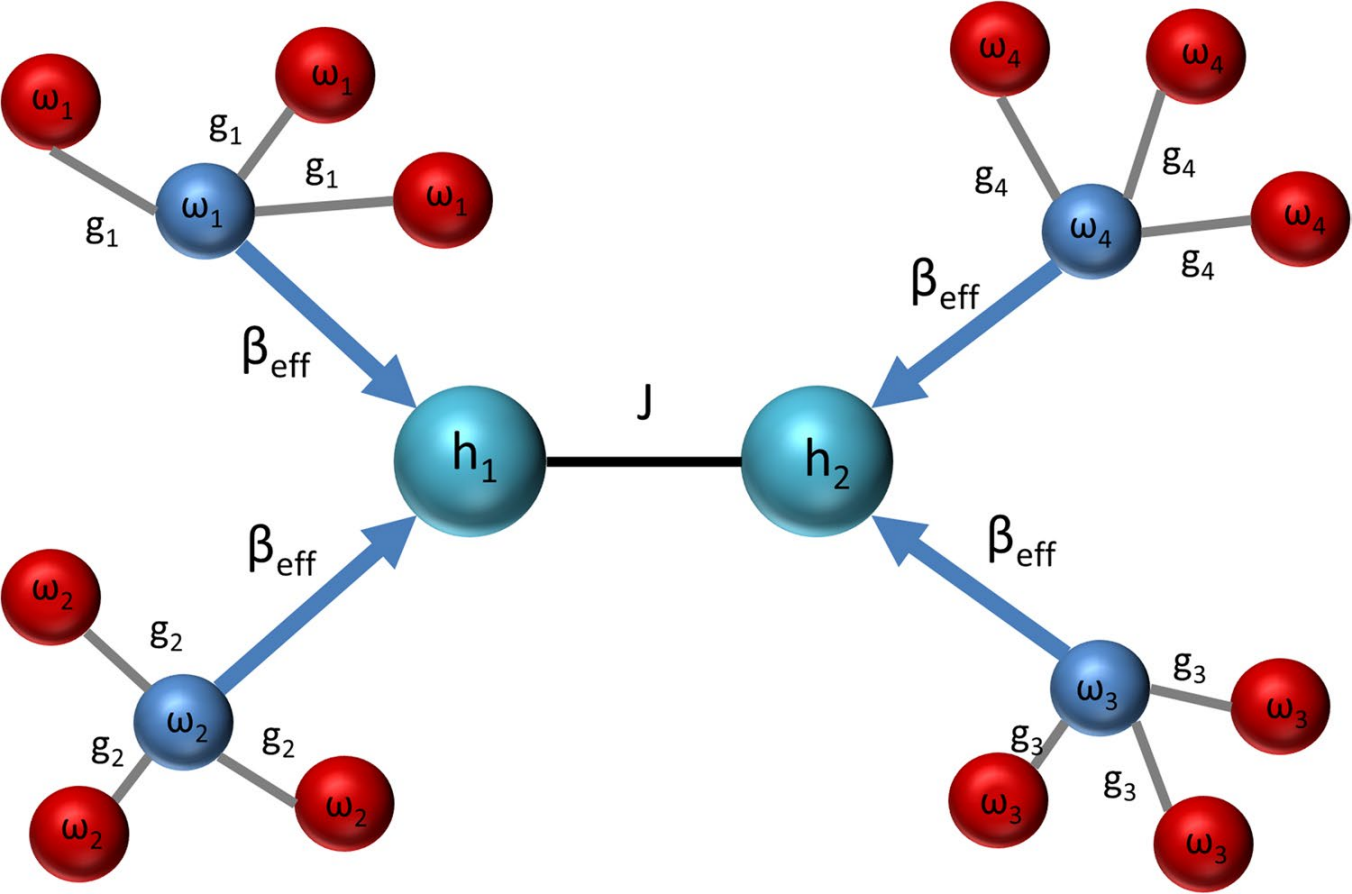
Sketch of a Markovian collision model cooling a two-spin longitudinal Ising model described by the Hamilonian in Eq. (16), with coupling strength *J*, using four spin-star quantum refrigerators labeled with $i=1 \ldots 4$. Central qubits of the refrigerators are the refrigerants at effective inverse temperature $\beta _{\text {eff}}$. Central qubits are not resonant with the Ising model qubits, whose energy gaps are denoted by $h_1$ and $h_2$, instead, they are resonant with the transition frequencies $\omega _i$ ($i=1 \ldots 4$) of the Ising model. Spin-star model has longitudinal and homogeneous couplings $g_i$.

Figure 4 shows a case where a two-qubit system is thermalized with the collision model. Our idea is to use the central qubits of spin-star systems as refrigerants (cooling fluid) in a refrigerator. Therefore, these central qubits are further coupled to another many-body system, again in the relatively hotter, same, joint environment. The refrigerator consists of the central qubit refrigerants (system B) and a target many-qubit system (system C) to be cooled while the ancilla qubits surrounding the central qubits cannot be considered as a part of the refrigerator in the cycle studied in the previous section because their mere purpose is the preparation of cold central qubits to be used in the refrigerator and the central qubits do not interact with the ancilla qubits during the collisions with the system C. For the case of collective cooling where all spins of system A interact with system C to cool it down, which will be studied in the next section, the refrigant would be the system A in its entirety. Also, we need to emphasize that, although the spin-star model can be implemented either as a classical or quantum system, the refrigerator needs quantum descriptions of the constituent refrigerants and target systems with discrete energy levels to generate the dynamics of a many-body quantum open system^25^ in a cold bath leading to its refrigeration. For this reason, we call our proposal a quantum refrigerator.

The system Hamiltonian in Fig. 4 is taken to be a longitudinal Ising model

16 $$\begin{aligned} {{\hat{H}}_\text {system}=\sum _{i=1}^2 h_i{\hat{\sigma }}_{z,i}+J{\hat{\sigma }}_{z,1}{\hat{\sigma }}_{z,2},} \end{aligned}$$

which gives four transition frequencies $\omega _i$^25^. Here $h_i$ with $i=1,2$ are the resonant frequencies of the system qubits, and *J* is the Ising coupling coefficients. It is then sufficient to collide each system qubit with two-bath qubits at different $\omega _i$. Most choices for the refrigerant qubit-system interaction Hamiltonian in the collisions lead to the master equation we derive in the SI appendix with the following condition: If we write the interaction Hamiltonian in the form $\sum _i {\hat{A}}_i \otimes {\hat{B}}_i$ where the operators act on a system qubit and the ancilla qubit respectively, there must be at least one operator ${\hat{A}}_i$ which does not commute with the many-body system Hamiltonian so that it can generate energy transitions with a corresponding Lindblad dissipator in the master equation.

In the present case, where our purpose is to cool down the system, the bath qubits are the central qubits coming out of the spin-star refrigerators at the third stage of the refrigeration cycle described in the previous section. Different spin-star refrigerators at different $h_i\equiv \omega _i /2$ should be adjusted to cool down their central qubits to the same $T_\text {eff}$ by using different $g_i$ [cf. Eq. (4)]. We outline the derivation of a Lindblad master equation for the collision model depicted in Fig. 4 in the SI appendix as an example of how the master equation corresponding to the open system dynamics generated by the collision model is derived for an arbitrary many-body system and we proceed to explain two different ways to increase the efficiency of the refrigeration cycle for the remainder of the main text.

### Final state of ancilla qubits and using them to enhance cooling efficiency

So far, we were only interested in the central qubit (system B) and traced out the ancilla qubits of system A in all of our calculations. We also defined the efficiency in Eq. (9) by excluding the energy change in the ancilla qubits. This may be a drawback for our proposal for large numbers of ancilla qubits and cooling to very cold temperatures because the work cost of the cycle in Eq. (15) is roughly proportional to the number of ancilla qubits while the energy extracted from the central qubit gets more or less saturated in very cold temperatures. As a workaround to this problem, we propose two possible uses of the ancilla qubits to increase the cooling efficiency. The first one is to use them in collisions with the many-qubit system for a cooperative effect and the second one is to use them in a heat engine cycle to help with the work required for running the spin-star refrigerators.

#### Cooperative cooling with ancilla qubits

**Figure 5:**
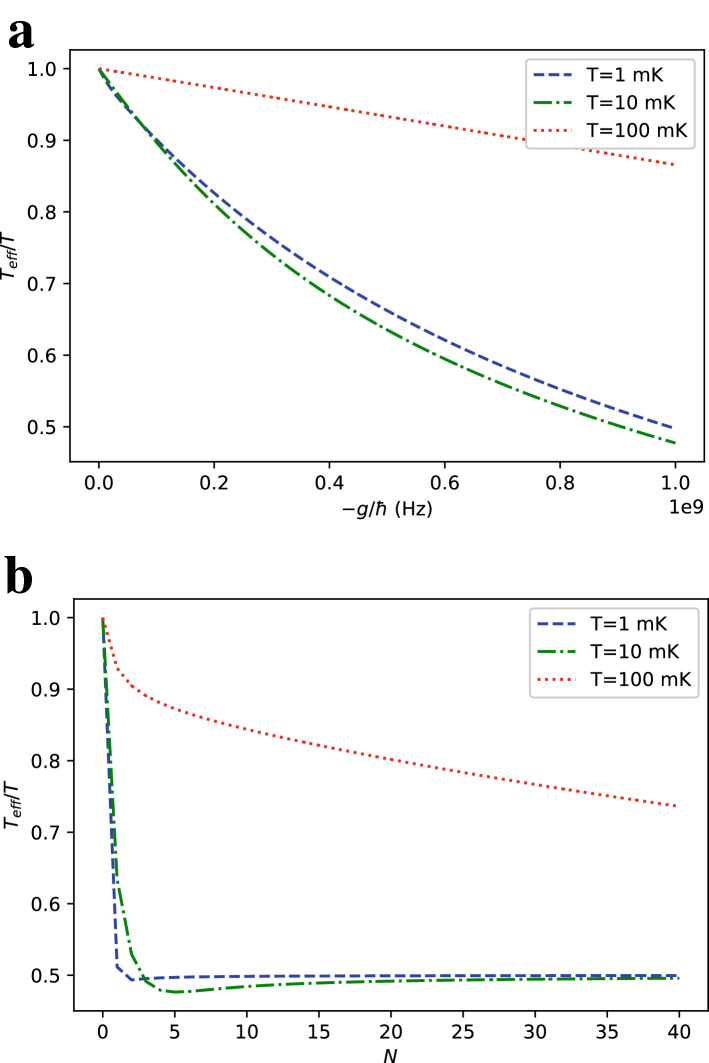
Ratio of the effective temperature $T_\text {eff} = 1/k_B \beta _\text {eff,whole}$ of the whole spin-star system after turning off its Ising interactions defined in Eq. (18) to the environment temperature *T* as a function of (**a**) interaction strength *g* with $N=6$ and (**b**) number of ancilla *N* qubits with $g=-h$. We take $h=1$ GHz.

Let’s consider using the ancilla qubits together with the central qubit as the refrigerant of the spin-star refrigerator. The cooling dynamics of our scheme is described by a Markovian master equation with additive Lindblad dissipators for simultaneous collisions. When all the uncoupled qubits of the spin-star system in the third stage of the refrigerator cycle collide with a qubit of the target system simultaneously, the resulting the master equation would be a straightforward generalization of the master equation derived for the case of only the central qubit used as refrigerant. The coefficients of two Lindblad dissipators in the master equation derived in the SI appendix responsible for heating and cooling become the sum of excited and ground state populations of the spin-star qubits, respectively. Accordingly, the multi-qubit system relaxes to a thermal state at temperature $T_\text {eff,whole}$ which now depends on *N*.

We can calculate $N_e$ and $N_g$ for a given set of spin-star qubits by using $N_e+N_g = N + 1$ and $N_e-N_g=\langle {\hat{S}}_z\rangle$ where ${\hat{S}}_z = \sum _{n=0}^{N} {\hat{\sigma }}_{z,n}$ and

17 $$\begin{aligned} <{\hat{S}}_z > = \frac{-1}{\beta } \frac{\partial \ln Z_{\text {tot}}}{\partial h}= & {} \frac{-2^N}{Z_{\text {tot}}}(e^{\beta h}\cosh ^N (\beta (h-g))- e^{-\beta h}\cosh ^N (\beta (h+g))\nonumber \\&+N(e^{-\beta h}\sinh (\beta (h+g))\cosh ^{N-1} (\beta (h+g))+e^{\beta h}\sinh (\beta (h-g))\cosh ^{N-1} (\beta (h-g)))). \end{aligned}$$

$T_\text {eff,whole}$ is then given by

18 $$\begin{aligned} \beta _{\text {eff,whole}} = \frac{1}{k_B T_{\text {eff,whole}}} = \frac{1}{2h} \ln \left( \frac{N+1-<{\hat{S}}_z>}{N+1+<{\hat{S}}_z>}\right) . \end{aligned}$$

Cooling of the many-qubit system with transition frequencies $\omega _i$ requires collisions with sets of spin-star refrigerant qubits with $2h_i=\omega _i$. Each spin-star cluster, associated with a different $\omega _i$, must be at the same $T_\text {eff,whole} = 1/k_B \beta _\text {eff,whole}$, which can be satisfied by using $g_i$. Under this condition, $T_\text {eff,whole}$ will be the temperature of the multi-qubit system in a steady state due to the repeated simultaneous collisions with the sets of the spin-star qubits. Figure 5 shows $T_\text {eff}$ for an example, where $\omega _i = 2$ GHz so that $h_i \equiv h = 1$ GHz for a particular set of spin-star qubits. For a target $T_\text {eff}$ one can determine the required $g_i \equiv g$ from Fig. 5. Comparison of Fig. 2 with Fig. 5 indicates that using only central qubits as the refrigerants of the spin-star quantum refrigerators yields colder $T_\text {eff}$ for the many-body system.

**Figure 6:**
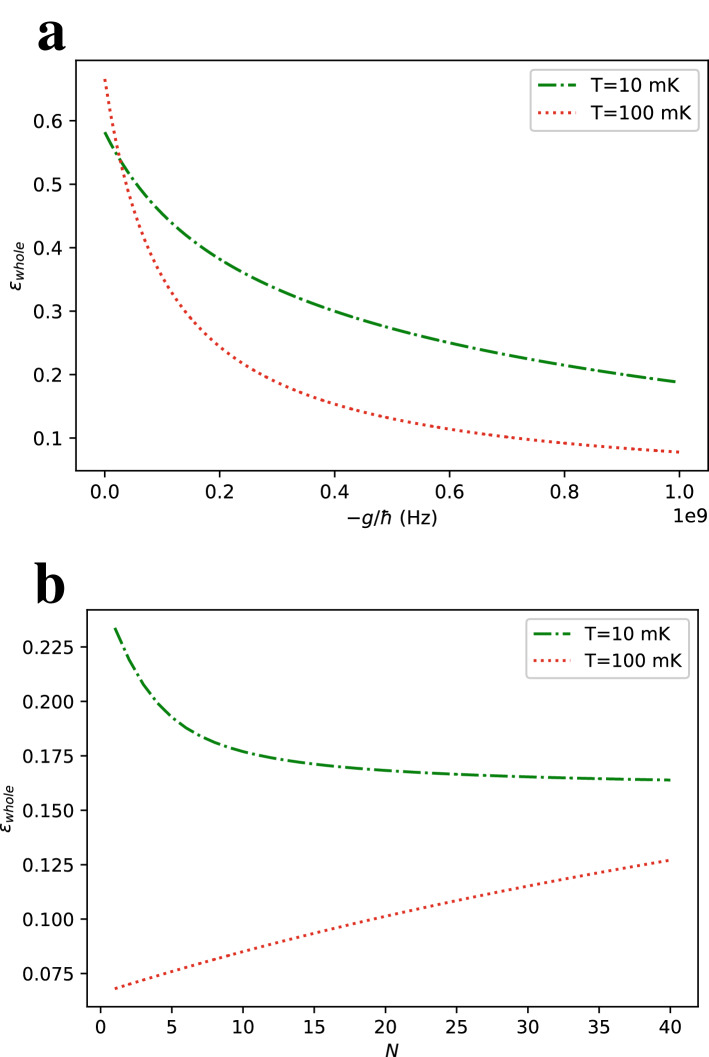
Efficiency $\varepsilon _{\text {whole}}$ defined in Eq. (19) as a function of (**a**) interaction strength *g* with $N=6$ and (**b**) number of ancilla qubits *N* with $g=-h$ at different environment temperatures *T*. We take $h = 1$ GHz.

As a concrete example of how limited this proposal is in terms of cooling the target many-body system, we observe from Fig. 5 that the ratio does not get significantly lower than 0.5 for reasonable coupling strengths and unrealistically large numbers of ancilla qubits. We expect the relative advantage of using the whole spin star qubits should lie in the cooling efficiency. We define the efficiency of the cycle for cooperative cooling as

19 $$\begin{aligned} \varepsilon _{\text {whole}} = \frac{E_0-E_{3}}{W_{\text {cycle}}} \end{aligned}$$

where the numerator is the total energy loss of the spin-star system instead of the energy loss of the central qubit only as in Eq. (9) and the quantities $E_0$ and $E_3$ take the values calculated in Eqs. (10) and (14). The resulting efficiency with all the spin-star qubits for different *g* and *N* is plotted in Fig. 6a and b respectively, showing an anticipated increase in efficiency for all *N* and *g* compared to Eq. (9). By a comparison with Fig. 3, the efficiency $\varepsilon _{\text {whole}}$ is several times higher than its counterpart $\varepsilon$ without the contribution of the ancilla qubits for most of the parameter choices. The increase of efficiency with the use of ancilla qubits is found to be particularly high in Fig. 6b, up to an order of magnitude for $T=10~\text {mK}$ which corresponds to the regime $h~ \text {~{}}~ k_B T/\hbar$ and high numbers of ancilla qubits.

Based on our numerical results, we can conclude that the cooperative cooling with ancilla qubits always increases the efficiency but it significantly increases the minimum achievable effective temperature especially for high numbers of ancilla qubits compared to the case where only the central qubit is used for cooling of the target many-body system. However, this trade-off between achieving cooling to very cold temperatures and efficiency, which manifests itself as the dynamical third law of both classical^48^ and quantum^49^ thermodynamics, is the main challenge of all refrigeration schemes and it persists with our proposal. Also, cooperative cooling allows makes the thermalization of the target many-body system at the temperature $T_\text {eff,whole}$ faster and more robust against the inevitable effects of the environment on the many-body system.

To address the trade-off between reaching very low temperatures and refrigeration with high efficiency, we also consider discarding some of the ancilla qubits. For this purpose, we calculate the expectation of the operator defined as ${\hat{S}}'_z = \sum _{n=1}^{N} {\hat{\sigma }}_{z,n}$ by expressing the total spin-star Hamiltonian and its partition function as

20 $$\begin{aligned} {\hat{H}}_{\text {Ising}}= & h_0~{\hat{\sigma }}_{z,0} + h_1\sum _{n=1}^{N}{\hat{\sigma }}_{z,n} + g~{\hat{\sigma }}_{z,0}\sum _{n=1}^{N}{\hat{\sigma }}_{z,n}, \end{aligned}$$

21 $$\begin{aligned} Z_{\text {tot}}= & 2^N (e^{-\beta h_0}\cosh ^N(\beta (g+h_1))+e^{\beta h_0}\cosh ^N(\beta (h_1-g))) . \end{aligned}$$

We take $h_0=h_1=h$, which gives

22 $$\begin{aligned} <{\hat{S}}'_z> &= \frac{-1}{\beta } \frac{\partial \ln Z_{\text {tot}}}{\partial h_1}\nonumber \\&\quad = \frac{-2^N N}{Z_{\text {tot}}}(e^{-\beta h}\sinh (\beta (h+g))\cosh ^{N-1}(\beta (h+g))+e^{\beta h}\sinh (\beta (h-g)) \cosh ^{N-1} (\beta (h-g))). \end{aligned}$$

As the spin-star Hamiltonian is symmetric with respect to permutations of ancilla qubits, all of the ancilla qubits have the same ground and excited populations, so that we can calculate the effective temperature of ancilla spins similarly to Eq. (18) as

23 $$\begin{aligned} \beta _{\text {eff,ancilla}} = \frac{1}{k_B T_{\text {eff,ancilla}}} = \frac{1}{2h} \ln \left( \frac{1-\frac{<{\hat{S}}'_z>}{N}}{1+\frac{<{\hat{S}}'_z>}{N}}\right) . \end{aligned}$$

**Figure 7:**
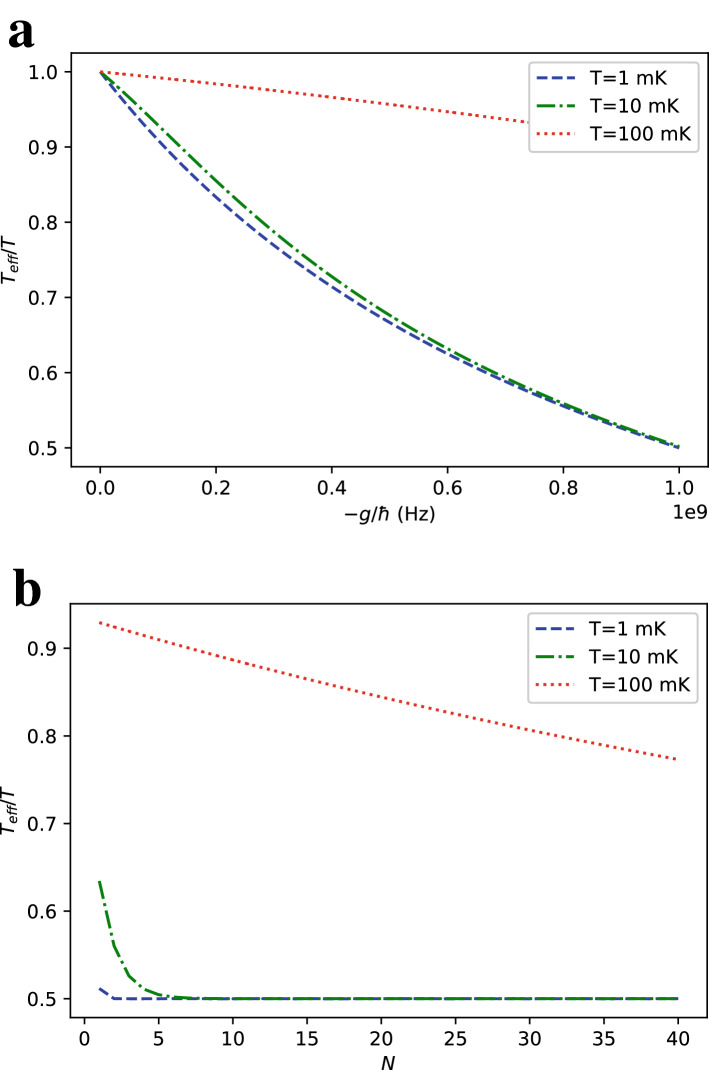
Ratio of the effective temperature of the ancilla qubits $T_{\text {eff}} = 1/k_B \beta _{\text {eq,ancilla}}$ after turning off Ising interactions defined in Eq. (23) to the environment temperature *T* as a function of (**a**) interaction strength *g* with $N=6$ and (**b**) number of ancilla qubits *N* with $g=-h$. We take $h = 1$ GHz.

The resulting effective ancilla temperature is plotted in Fig. 7. It is always higher than the center qubit effective temperature in Fig. 2 except for the trivial case of $N=1$ ancilla qubits. Therefore, the excited population of the ancilla qubits is always greater or equal to the excited population of the central qubit.

Now, we can define an effective temperature of collective cooling when a number $n \le N$ of the ancilla qubits are used as

24 $$\begin{aligned} \beta _{\text {eff,n}} = \frac{1}{k_B T_{\text {eff,n}}} = \frac{1}{2h} \ln \left( \frac{n+1-\frac{n<{\hat{S}}'_z>}{N}+\tanh (\beta _{\text {eff}}h)}{n+1+\frac{n<{\hat{S}}'_z>}{N}-\tanh (\beta _{\text {eff}}h)}\right) . \end{aligned}$$

As we are able to see $|<{\hat{S}}'_z>/N|<\tanh (\beta _{\text {eff}}h)$ by comparing Figs. 2 and 7, we also have $\beta _{\text {eff}}>\beta _{\text {eff,n}}>\beta _{\text {eff,ancilla}}$. We can define the efficiency of the refrigeration cycle for the case of discarding some ancillae by ignoring the energy taken from these qubits, but the result is obvious: This efficiency would be between Eqs. (9) and (19).

#### Ancilla qubits used as a cold bath for a quantum heat engine

**Figure 8:**
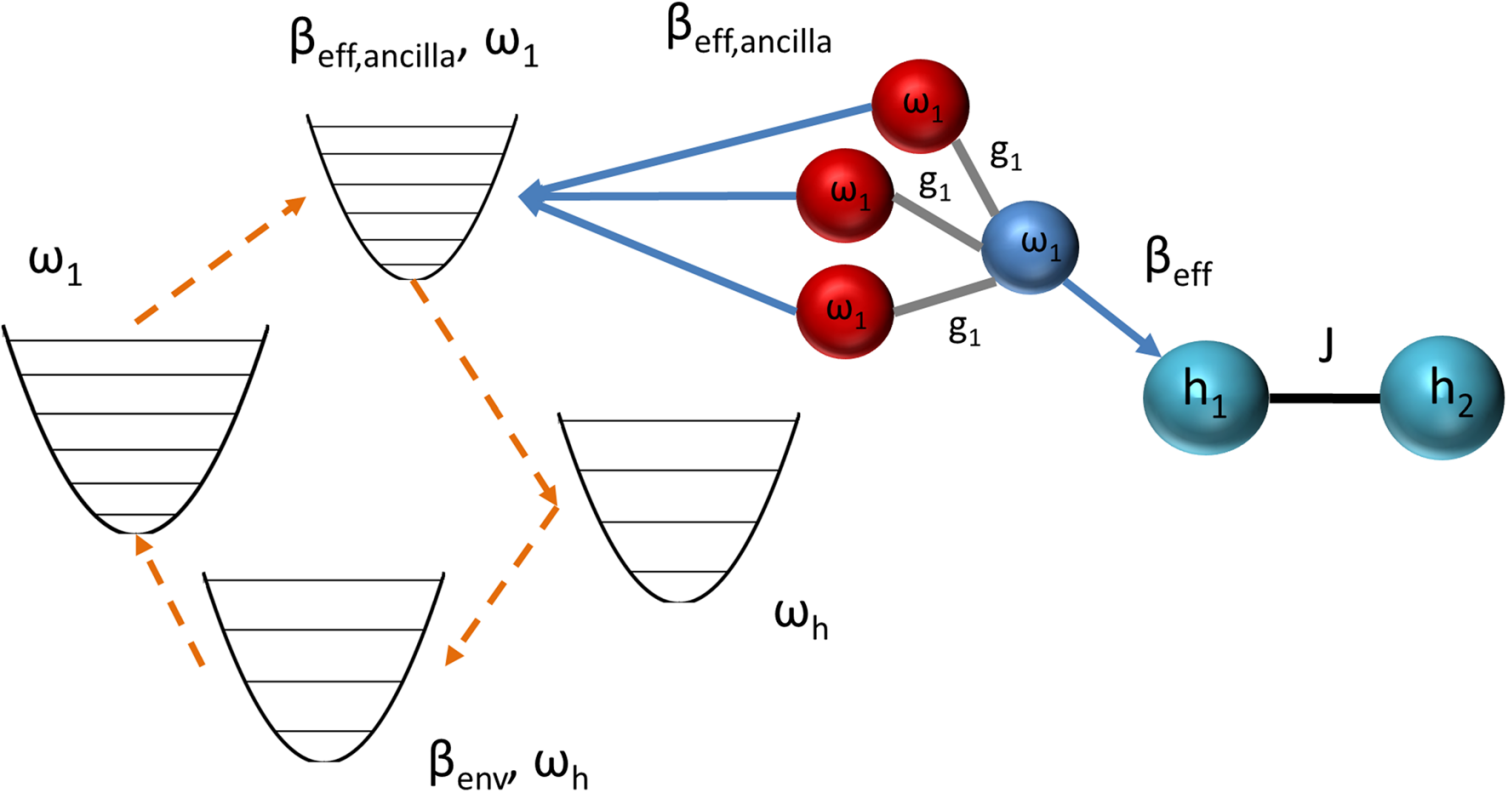
Simplified sketch of an improved quantum many-body refrigerator which uses the cold ancilla qubits in a quantum Otto cycle to mimic a cold reservoir and harvest work from the environment, which is the hot reservoir of the cycle. The central qubit is used to cool down the system C along with other refrigerant qubits from other spin-star systems (omitted in the figure for simplicity) with its effective temperature $\beta _{\text {eff}}$ from Eq. (4) while the ancilla qubits at effective temperature $\beta _{\text {eff,ancilla}}$ from Eq. (23) are used to mimic a cold reservoir for a quantum Otto cycle in which the working medium is a separate quantum harmonic oscillator as shown on the left side of the figure. The spheres in light blue and the sphere in dark blue represent the system C and the system B respectively while the spheres in red are the ancilla qubits of the system A. The arrows in blue represent the collisions which cool down the target system pointed by the arrow. The dashed arrows in orange represent the evolution of a single harmonic oscillator throughout its Otto cycle.

Although using all qubits allows reasonable efficiency values for a specific temperature range, we propose another way of using the ancilla qubits of system A to increase efficiency. As the center qubit’s effective temperature gets lower with the increasing number of ancilla qubits while the effective temperature of ancilla qubits do not, we suggest that the center qubit can be used to cool down a many-body system to a very cold temperature. Remarkably, ancilla qubits are also in a thermal state colder than the environment. Hence, instead of sitting idle while the system B interacts with the system C, ancilla qubits can be utilized as cold bath for an Otto engine which provides the work to the refrigerator (see Fig. 8). Such an Otto engine with the the ancilla qubits as cold bath would “recycle” some of the work spent in the refrigeration cycle after the Ising interaction of the spin-star system is turned off in a thermalized state, which corresponds to the interval between the third and fourth steps of the refrigeration cycle.

Similar to the many-body cooling discussed in the previous section, the interaction of the ancilla qubits with this engine must take place in a timescale much smaller than the relaxation time of the qubits to the environment temperature. For this proposal, the efficiency would depend on the type of engine in question, but we can give a reasonable definition of efficiency

25 $$\begin{aligned} \varepsilon _{\text {re}} = \frac{h(\tanh (\beta _{\text {eff,center}} h)-\tanh (\beta h))}{W_{\text {cycle}}-W_{\text {engine}}} \end{aligned}$$

based on the efficiency definition in Eq. (9) without the contribution of the engine.

To gain insight into how large $W_{\text {engine}}$ can get, it is useful to calculate the effective temperature of the environment qubits after tracing out the center qubit by finding the ratio of the total ground and excited populations of the ancilla qubits. As all ancilla qubits are at the same effective temperature $\beta _{\text {eff,ancilla}}$, their collective effective temperature is also the same. This argument also applies to cases where some of the ancilla qubits are discarded. Fig. 7 shows the equilibrium temperature when all of the ancilla qubits in the spin-star system is used for collisions with the engine as its artificial cold reservoir. The plot is somehow similar to Fig. 5 with center qubit included.

Now that we have some qualitative results on the effective temperature of ancilla spins, we can make a more detailed comment on a possible engine working with the ancilla spins and its work production. As an analytically tractable^50^ and experimentally realizable model^51^, we propose to use a quantum Otto engine using a harmonic oscillator as its working medium. For this engine, the environment would be the hot bath at the inverse temperature $\beta$ and the ancilla spins would be the cold bath at the inverse temperature $\beta _{\text {eq,ancilla}}$ using our previously proposed collision model^25^.

As the thermalization of a system happens asymptotically with the number of collisions diverging to infinity, we assume that the number of ancilla spins *N* is sufficiently large so that they are able to bring the harmonic oscillator to their effective temperature with negligible deviation. To summarize the quantum Otto cycle, the harmonic oscillator thermalized at the inverse temperature $\beta$ and the frequency $\omega _h$ is adiabatically driven to a lower frequency $\omega _c$, leading to a work extraction. Then, the harmonic oscillator is brought to the inverse temperature $\beta _{\text {eq,ancilla}}$ by collisions with ancilla spins, and it is driven back to the frequency $\omega _h$, taking some work from outside and completing the cycle. However, we cannot suppress the effects of the environment at the inverse temperature $\beta$ during the adiabatic strokes in an experimental realization of this engine, and we need to implement adiabatic strokes in short times so that the effect of the environment on these steps can be neglected, making these strokes strongly non-adiabatic and reducing the efficiency^52^. Another widely studied modification of this cycle is to introduce squeezing in the hot reservoir^53^, which is shown to exceed the Carnot efficiency^54^ and even reach a unity efficiency for some choices of engine parameters^55^.

## Discussion

In summary, we proposed a scheme for cooling a multi-qubit system using a set of refrigerant qubits prepared by spin-star systems. We found that the central qubits of the spin-star systems we introduce are in a canonical Gibbs state if they are coupled to the ancilla qubits of the spin-star system longitudinally. Moreover, this Gibbs state is characterized by an effective temperature colder than the environment if the spin-star system consists only of longitudinal spins and ferromagnetic interactions. Analytical calculations showed that the central spin temperature monotonically decreases with the strength of Ising interactions. For asymptotically strong interactions, the central spin temperature scales by a factor of $1/(N+1)$. We determined that bringing the effective temperature of the central spin by an order of magnitude with respect to its environment is possible using the typical range of parameters from superconducting circuit systems. We analyzed the efficiency of a simple refrigeration cycle, discussed how the refrigerant qubits prepared in each cycle can cool a many-body system to their effective temperature; besides, we suggested two different uses of ancilla spins in the refrigeration cycle to further increase the efficiency. Our results for a scalable and efficient cooling of a many-body system using few qubit artificial environments can be significant for compact implementations of quantum computation, metrology, or simulator technologies^56,57^.

## Methods

We do not have any experimental results in our work. All of the plots are based on our analytical results and they are reproducible from the relevant equations. We produced the plots using Matplotlib library of Python. The numerical results plotted in the main text are obtained using NumPy library of Python and the results in the SI appendix are obtained using QLib library^58^ for Matlab.

## Supplementary Information

Supplementary Information.
