# Supplementary material for: Few-qubit quantum refrigerator for cooling a multi-qubit system
Source: Sci Rep. 2021 Jun 21;11:12981. doi: 10.1038/s41598-021-92258-0 (PMC8217472; doi:10.1038/s41598-021-92258-0)
Supplement: Supplementary file 1 — Supplementary Information. [file 41598_2021_92258_MOESM1_ESM.pdf]

Supplementary Information for "Few-qubit quantum refrigerator for cooling a multi-qubit system"

Onat Arısoy[1] **and Özgür E. Müstecaplıoğlu**[2,*]

[1]Institute for Physical Science and Technology, University of Maryland, College Park, Maryland 20742, USA
[2]Department of Physics, Koç University, Sarıyer, İstanbul, 34450, Turkey
[*]omustecap@ku.edu.tr

ABSTRACT

1 Spin-star quantum refrigerator with Heisenberg model interactions

We argue here that a previously studied spin-star quantum heat engine model[1], different from our longitudinal Ising couplings due to the additional Heisenberg type transverse spin component interactions, can operate as a refrigerator as well for an appropriate choice of parameters. While the Ising interaction is suitable for an analytical study with classical statistical mechanics methods, we are not aware of any practical way of calculating the central qubit's density matrix with an arbitrary number of ancilla qubits. For this reason, we will restrict ourselves to show numerical results with a small number of ancilla qubits. The Hamiltonian of the whole system with Heisenberg interaction is given by

$$\hat{H}_{\text{Heisenberg}} = h\sum_{n=0}^{N}\hat{\sigma}_{z,n} + g\sum_{i=x,y,z}\sum_{n=1}^{N}\hat{\sigma}_{i,0}\hat{\sigma}_{i,n}. \tag{1}$$

This choice corresponds to $\lambda = 1$ for the previously studied engine model[1] for which the authors report a faster than linear increase of the ratio $T_{\text{eff}}/T_{\text{bath}}$ with respect to increasing number of environment qubits. Contrary to the assumptions of Ref.[1] and following our results for Ising interaction, we study the regime $g < 0$ in Eq. (1).

Fig. 1a shows our numerical results for the ratio of effective temperature to the environment temperature for Heisenberg interaction. Our simulations for 2 and 4 ancilla qubits yielded very similar results, and we did not observe any significant change in the effective temperature of the central qubit. The Ising model is a better choice to significantly lower the effective temperature, especially with large numbers of ancilla qubits. The relatively poor cooling with Heisenberg interaction in our spin-star quantum refrigerator agrees with previous works reporting specific heat anomalies and non-zero excitation probability at zero temperature due to system-bath entanglement[2–4]. Quantum coherence and entanglement work against cooling in our scheme based upon equilibration with a single effective bath generated by qubit-qubit collisions. Remarkably, using multiple reservoirs and non-equilibrium scenarios, one could enhance the cooling efficiency by exploiting quantum coherence and correlations[5–7].

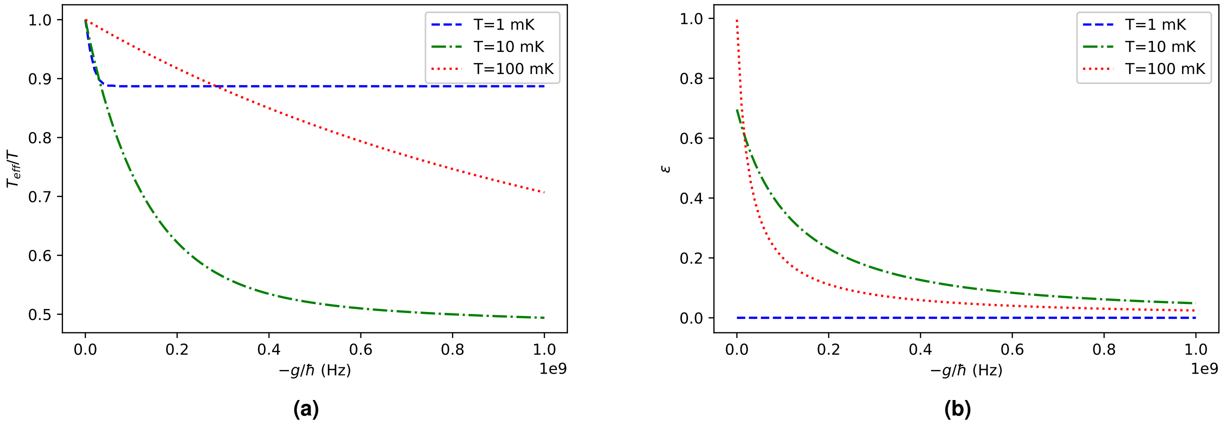

Figure 1. (a) Ratio of the effective T_{eff} temperature of the central qubit to the environment temperature T and (b) cooling efficiency ε with a Heisenberg spin-star model for $N = 6$ ancilla qubits at different interaction strengths g. We take $h = 1$ GHz.

Fig. 1b shows the efficiency of our refrigerator cycle described in the main text with different temperatures and different interaction strengths for six ancilla qubits for Heisenberg interactions. Heisenberg interaction seems to be much more efficient at first sight; however, we need to emphasize that a poor refrigeration performance accompanies its high efficiency.

2 Derivation of a Lindblad master equation for our collision model

The derivation of the Markovian master equation in Lindblad form for a many-body collision model[8] is based upon the set of standard assumptions of open quantum systems weakly coupled to large reservoirs[9]. Starting with the Liouville-von Neumann equation for the system and environment coupling Hamiltonian $\hat{H}_I(t)$ in the interaction picture

$$i\hbar\frac{\partial\rho}{\partial t} = [\hat{H}_I(t), \rho], \tag{2}$$

we integrate it over time. This gives an expression of $\rho(t)$ as an integration over a function of its past values. As we integrate Eq. (2) over time, we can plug in the expression for $\rho(t)$ in its right-hand side arbitrarily many times to derive different expressions for $\rho(t)$. Doing this twice with the assumption $\text{Tr}_B([\hat{H}_I(t), \rho(0)]) = 0$ gives

$$\frac{d}{dt}\rho_s(t) = -\int_0^t ds \text{Tr}_B([\hat{H}_I(t), [\hat{H}_I(s), \rho(s)]]). \tag{3}$$

At this point, assuming negligible change in the bath and system states and neglecting the system-bath entanglement to the resulting equation yields the following.

$$\frac{d}{dt}\rho_s(t) = -\int_0^t ds \text{Tr}_B([\hat{H}_I(t), [\hat{H}_I(s), \rho_s(s) \otimes \rho_b]]) \tag{4}$$

This step is conventionally justified with a large, or infinite, bath coupled to the system for a long time[9]. These assumptions are in sharp contrast with short-time collisions with a single two-level system. Nevertheless, we will outline how one could ignore the finite time effects in the master equation under certain conditions[8]. To do that, we proceed with the derivation of master equation for a large bath[9] and we will emphasize where this derivation takes a different turn when it comes to collisions with two-level systems. Our next step is to apply Markov approximation and replace $\rho_s(s)$ with $\rho_s(t)$ in Eq. (4) to obtain the Redfield equation. For the case of short-time collisions, this can be justified by assuming that the change of the system state is too little to have an effect on its future evolution. Then, we extend the integration in Eq. (4) from t to infinity as $H_I(t)$ is zero for times outside the collision interval. This gives the following.

$$\frac{d}{dt}\rho_s(t) = -\int_0^\infty ds \text{Tr}_B([\hat{H}_I(t), [\hat{H}_I(t-s), \rho_s(t) \otimes \rho_b]]). \tag{5}$$

We introduce the interaction Hamiltonian in the Schrödinger picture with the following decomposition

$$\hat{H}_I = \sum_{\alpha} \hat{A}_\alpha \otimes \hat{B}_\alpha \tag{6}$$

such that the Hermitian operators $\hat{A}_\alpha$ and $\hat{B}_\alpha$ act on the system and the bath respectively. After further decomposing the operators $\hat{A}_\alpha$ into operators $\hat{A}_\alpha(\omega)$ that generate energy transitions with frequency ω, Eq. (5) becomes

$$\frac{d}{dt}\rho_s(t) = \sum_{\omega,\omega'} \sum_{\alpha,\beta} e^{it(\omega'-\omega)} \Gamma_{\alpha\beta}(\omega) \left(\hat{A}_\beta(\omega)\rho_s(t)\hat{A}^\dagger_\alpha(\omega') - \hat{A}^\dagger_\alpha(\omega')\hat{A}_\beta(\omega)\rho_s(t) \right) + \text{h.c.} \tag{7}$$

where $\Gamma_{\alpha\beta}(\omega)$ is the one-sided Fourier transform of the reservoir correlation functions which is defined as the following with the interaction picture version of operators $\hat{B}_\alpha$

$$\Gamma_{\alpha\beta}(\omega) = \int_0^\infty ds e^{is\omega} \text{Tr}_B(\hat{B}^\dagger_\alpha(t)\hat{B}_\beta(t-s)). \tag{8}$$

At this point, the specification of the system, bath and interaction Hamiltonians is needed to proceed further. Following our previous work[8], we first work out the thermalization of a two-level system with collisions to show that each colliding ancilla qubit needs to be resonant with one of the energy transitions of the system, then we generalize our results to many-body case.

2.1 Thermalization of a two-level system with collisions

For this case, the system, bath and interaction Hamiltonians are given by

$$\hat{H}_S = h_s \hat{\sigma}_z \tag{9}$$

$$\hat{H}_B = \sum_{n=1}^{N} h_b \hat{\sigma}_{zn} \tag{10}$$

$$\hat{H}_I = \sum_{n=1}^{N} g_n(t) \hat{\sigma}_x \otimes \hat{\sigma}_{xn} \tag{11}$$

where the subscript indicates that the operator acts on n^{th} ancilla and the operator acts on the target two-level system when there is no subscript.

Knowing the collision period and duration, the time-dependent interaction strengths are defined as

$$g_n(t) = \theta(t-(n-1)\tau_p)\theta((n-1)\tau_p + \tau_c - t)g \tag{12}$$

where θ denotes the Heaviside step function.

As the interaction with each ancilla is in the form of Eq. (6) with a single element in the sum and it is straightforward to show that the cross-correlation between different ancillae is zero, Eq. (7) can be simplified.

$$\frac{d}{dt}\rho_s(t) = \sum_{\omega,\omega'} \sum_{n=1}^{N} e^{it(\omega'-\omega)} \Gamma_n(\omega,t) \left(\hat{A}_n(\omega)\rho_s(t)\hat{A}^\dagger_n(\omega') - \hat{A}^\dagger_n(\omega')\hat{A}_n(\omega)\rho_s(t) \right) + \text{h.c.} \tag{13}$$

The bath correlation spectrum functions $\Gamma_n(\omega,t)$ are worked out in our previous work[8] and we simply report here the end result.

$$\Gamma_n(\omega,t) = -ig^2 \left(\frac{\rho^n_{ee}(\exp(i(t-(n-1)\tau_p)(\omega+2h_b))-1)}{\omega+2h_b} + \frac{\rho^n_{gg}(\exp(i(t-(n-1)\tau_p)(\omega-2h_b))-1)}{\omega-2h_b} \right), \quad \omega \neq \pm 2h_b. \tag{14}$$

$$\Gamma_n(\omega,t) = g^2(\rho^n_{ee}(t-(n-1)\tau_p)) - \frac{i\rho^n_{gg}(\exp(i(t-(n-1)\tau_p)(\omega-2h_b))-1)}{\omega-2h_b}, \quad \omega=-2h_b \tag{15}$$

$$\Gamma_n(\omega,t) = g^2(\rho^n_{gg}(t-(n-1)\tau_p)) - \frac{i\rho^n_{ee}(\exp(i(t-(n-1)\tau_p)(\omega+2h_b))-1)}{\omega+2h_b}, \quad \omega=2h_b \tag{16}$$

We will restrict ourselves to the case of $\omega = \pm 2h_b$ and $\tau_c \gg 1/\omega_b$ to introduce secular approximation and neglect the second terms of Eqs. (15) and (16). The approximate form of the bath correlation spectrum is the following[8].

$$\mathrm{Re}(\Gamma(\omega,t)) = g^2 \sum_{n=1}^{N} (\delta'(\omega-2h_b)\rho^n_{gg} + \delta'(\omega+2h_b)\rho^n_{ee})(t-(n-1)\tau_p)\theta(t-(n-1)\tau_p)\theta((n-1)\tau_p+\tau_c-t) \tag{17}$$

Here, the function $\delta'(\omega)$ is defined as one for $\omega=0$ and zero elsewhere to introduce our neglection of off-resonance terms in the equation. Then, the master equation for a two-level system becomes

$$\frac{d}{dt}\rho_s(t) = \mathrm{Re}(\Gamma(2h_s,t))(\hat{\sigma}_-\rho_s(t)\hat{\sigma}_+ - \frac{1}{2}\{\hat{\sigma}_+\hat{\sigma}_-,\rho_s(t)\}) + \mathrm{Re}(\Gamma(-2h_s,t))(\hat{\sigma}_+\rho_s(t)\hat{\sigma}_- - \frac{1}{2}\{\hat{\sigma}_-\hat{\sigma}_+,\rho_s(t)\}). \tag{18}$$

At this point, it is in order to reiterate the assumptions and approximations we made in order to proceed with the generalization to many-body systems. First, the two-level system must be in resonance with one of the transition frequencies of the system, the off-resonance case is numerically studied in our previous work[8] and it is shown that the open system dynamics is either negligibly slow or totally frozen in the off-resonance case. Second, the collisions must take much longer time than the inverse of the transition frequency in question and it must also satisfy $g\tau_c \ll 1$ in order to assert that the system and ancilla density matrices do not change significantly during a single collision. These assumptions give both a lower and a lower bound to the collision duration such that the Lindblad master equation we derived is valid.

The equilibration of the system at the temperature of ancilla qubits is straightforward to prove from the Kubo-Martin-Schwinger (KMS) condition for the bath correlation spectrum[8].

$$\frac{\mathrm{Re}(\Gamma_n(2h_s,t))}{\mathrm{Re}(\Gamma_n(-2h_s,t))} = \exp(2\beta h_s) = \frac{\rho^n_{gg}}{\rho^n_{ee}} = \exp(2\beta_b h_s) \tag{19}$$

2.2 Generalization to many-body systems

The master equation we derived in the previous section can still be generalized to systems with arbitrarily many energy levels by interpreting the master equation in the subspace spanned by states separated by the resonant frequency, as all the off-resonance terms are neglected by secular approximation. This leads to Lindblad dissipators in the following form for each collision

$$D(\hat{\sigma}_-,\hat{\sigma}_+,\rho_s) \quad \propto \quad (\rho^{\mathrm{bath}}_{gg}(\hat{\sigma}_-\rho_s(t)\hat{\sigma}_+ - \frac{1}{2}\{\hat{\sigma}_+\hat{\sigma}_-,\rho_s(t)\}) + \rho^{\mathrm{bath}}_{ee}(\hat{\sigma}_+\rho_s(t)\hat{\sigma}_- - \frac{1}{2}\{\hat{\sigma}_-\hat{\sigma}_+,\rho_s(t)\})), \tag{20}$$

where $\rho^{\mathrm{bath}}_{gg}$ and $\rho^{\mathrm{bath}}_{ee}$ are the ground and excited state populations of the colliding "bath qubit" (central, refrigerant, qubit of the spin-star system) whose resonance frequency ω_i coincides with one of the transition frequencies of the system. The jump operators $\sigma_\pm$ are for a system qubit. The density matrix of the many-qubit system is denoted by ρ_s.

Once the elimination of off-resonance terms is justified, the generalization to multiple transition frequencies is straightforward as the dissipators of collisions with different bath qubits are additive[8]. Each collision generates a term similar to Eq. (20), responsible for transitions between two states separated by the bath qubit's frequency. The collision model discussed in the main text as an example of many-body thermalization gives rise to the master equation

$$\frac{d}{dt}\rho_s \propto \sum_{i=1}^{2}\sum_{\omega_i}\left(\rho_{g,\omega_i}D(\hat{\sigma}^{\omega_i}_{-i},\hat{\sigma}^{\omega_i}_{+i},\rho_s) + \rho_{e,\omega_i}D(\hat{\sigma}^{\omega_i}_{+i},\hat{\sigma}^{\omega_i}_{-i},\rho_s)\right), \tag{21}$$

where $\hat{\sigma}^{\omega_i}_{\pm i}$ are the single-qubit transition operators for the i-th bath qubit at resonance frequency ω_i[8]. $\rho_{g/e,\omega_i}$ are the ground/excited state populations of the bath qubits with resonance frequencies ω_i. The additivity of Lindblad dissipators arising from different collisions allow simultaneous collisions for a much faster implementation on a large system.

The thermal state of the target multi-qubit system is the unique equilibrium point of the collisional master equation, Eq. (21), when the generated transitions connect all of the states of the system[10]. The Kubo-Martin-Schwinger (KMS) conditions for the resulting master equation show that the target system's equilibrium temperature is the same as that of the refrigerant qubits T_{eff} [8,9]. One situation that requires a more complicated explanation about the thermalization is the case of a many-body system with entangled energy eigenstates. In this case, the system component of the interaction can be decomposed into many energy transitions as that local operator needs to be expressed in the energy basis before applying the secular approximation. This leads to cross correlations among the energy transitions generated by the same collisions and corresponding additional terms in the master equation. As these additional terms do not come into play for the detailed balance of the the energy state populations, they do not change the equilibrium state; however, the positive definiteness of the bath correlation matrix needs to be checked in the presence of cross correlations in order to make sure that the master equation makes the system evolve towards its equilibrium state[8].

In summary, we conclude that a thermalizing master equation can describe the interaction of the central qubits with the many-qubit system for the system to evolve into a thermal equilibrium state with the refrigerant central qubits out of spin-star refrigerators.

Although not depicted in Fig. 4 of the main text, the effect of the environment at a temperature $T > T_{\text{eff}}$ during the collisions also needs to be considered in a real application. Despite this setback, an appropriate choice of collision times and strengths can still bring the target system to equilibrium at a temperature $T_{\text{eff}} < T_{\text{eq}} < T$ as the dissipators due to the environment and the refrigerant qubits are additive.

References

1. Türkpençe, D., Altıntaş, F., Paternostro, M. & Müstecaplıoğlu, Ö. E. A photonic carnot engine powered by a spin-star network. *Eur. Lett.* **117**, 50002, DOI: 10.1209/0295-5075/117/50002 (2017).
2. Jordan, A. N. & Büttiker, M. Entanglement energetics at zero temperature. *Phys. Rev. Lett.* **92**, 247901, DOI: 10.1103/PhysRevLett.92.247901 (2004).
3. Ingold, G.-L., Hänggi, P. & Talkner, P. Specific heat anomalies of open quantum systems. *Phys. Rev. E* **79**, 061105, DOI: 10.1103/PhysRevE.79.061105 (2009).
4. Hasegawa, H. Specific heat anomalies of small quantum systems subjected to finite baths. *J. Math. Phys.* **52**, 123301, DOI: 10.1063/1.3669485 (2011).
5. Mitchison, M. T., Woods, M. P., Prior, J. & Huber, M. Coherence-assisted single-shot cooling by quantum absorption refrigerators. *New J. Phys.* **17**, 115013, DOI: 10.1088/1367-2630/17/11/115013 (2015).
6. Brask, J. B. & Brunner, N. Small quantum absorption refrigerator in the transient regime: Time scales, enhanced cooling, and entanglement. *Phys. Rev. E* **92**, 062101, DOI: 10.1103/PhysRevE.92.062101 (2015).
7. Brunner, N. *et al.* Entanglement enhances cooling in microscopic quantum refrigerators. *Phys. Rev. E* **89**, 032115, DOI: 10.1103/PhysRevE.89.032115 (2014).
8. Arısoy, O., Campbell, S. & Müstecaplıoğlu, Ö. E. Thermalization of finite many-body systems by a collision model. *Entropy* **21(12)**, 1182, DOI: doi:10.3390/e21121182 (2019).
9. Breuer, H. P. & Petruccione, F. *The Theory of Open Quantum Systems* (Oxford University Press, Oxford, UK, 2002).
10. Shabani, A. & Neven, H. Artificial quantum thermal bath: Engineering temperature for a many-body quantum system. *Phys. Rev. A* **94**, 052301, DOI: 10.1103/PhysRevA.94.052301 (2016).
